# Sieving the weeds from the grains: an R based package for classifying archaeobotanical samples of cereals and pulses according to crop processing stages

**DOI:** 10.1007/s00334-024-01006-7

**Published:** 2024-08-20

**Authors:** Elizabeth Stroud, Glynis Jones, Michael Charles, Amy Bogaard

**Affiliations:** 1 School of Archaeology, University of Oxford, Oxford, UK; 2 Department of Archaeology, University of Sheffield, Sheffield, UK

**Keywords:** Crop-processing, Discriminant analysis, Weed seed attributes, R package, Cereal and pulse processing

## Abstract

The R package CropPro is an open-access resource to classify archaeobotanical samples as products and by-products of different stages of the crop processing sequence for large-seeded cereal and pulse crops in south west Asia, Europe and other Mediterranean regions. It builds on ethnographic research and analysis conducted by Jones (Plants and ancient man: studies in palaeoethnobotany. Balkema, Rotterdam, pp 43–61, 1984), (J Archaeol Sci 14:311–323, 1987), (Circaea 6:91–96, 1990) and a modified method by Charles (Environ Archaeol 1:111–122, 1998). CropPro provides functions, which allow users to construct triplots, to conduct discriminant analysis comparing archaeobotanical samples with ethnographic crop processing stages and to plot the discriminant analysis results. This paper provides two worked examples of the use of CropPro: the early medieval site of Stafford in the UK and the Bronze Age site of Tell Brak in Syria. These examples illustrate the use of the package for identifying crop-processing stages, and for assessing the relevance of taphonomic pathways other than crop processing.

**Supplementary Information:**

The online version contains supplementary material available at 10.1007/s00334-024-01006-7.

## Introduction

Understanding the crop processing stages represented by archaeobotanical remains is essential for identifying activity areas, seasonal activities, and storage protocols at early agricultural sites. The series of steps required to convert harvested crop material into clean grain has been recognized as one of the causes of variation in archaeobotanical samples (Dennell 1972, 1974, 1976; Hillman 1973). For this reason, determining the crop processing status of archaeobotanical samples is necessary in order to recognise the biases imposed by such activities on the composition of archaeobotanical samples, and to consider this bias during interpretation. This includes changes in the proportions of different weed species, which can be particularly important when using weed species as indicators of cultivation regimes (e.g. Bogaard et al. 2005).

Ethnobotanical studies on crop processing highlight how crop-processing sequences alter both the crop and weed composition of a sample (Hillman 1981; Jones 1984, 1987, 1990). Several archaeobotanists have conducted or used ethnographic research to understand the processing sequence of a range of crop species (see for example Hillman 1981, 1984a, 1985; Jones 1984; D’Andrea and Haile 2002; Peña-Chocarro and Zapata Peña 2003 for temperate cereals and pulses; Reddy 1997, 2003; Thompson 1998; Lundström-Baudais et al. 2002; Harvey and Fuller 2005 for millets and rice). Such research has been taken further, with the proportions and ratios of particular items within such ethnographic data used to infer the crop processing status of archaeobotanical material (see for example Hillman 1984b; Jones 1984, 1990). Jones (1984, 1987) used ethnographic data of the weed seed characteristics as a discriminant model, which provides a way of recognising the effect of crop processing on archaeobotanical samples. Ethnographic work, conducted on the Greek island of Amorgos in the 1980s laid the foundation for statistical models used to identify archaeobotanical samples as the products and by-products of different stages in the traditional crop processing sequence for large-seeded cereal and pulse crops in south west Asia, Europe, and other Mediterranean regions (Jones 1984, 1987). By collecting and characterising these (by-)products of processing, data were obtained for three different statistical models that allow a comparison between ethnographic and archaeobotanical data. Although the processing of these crops is applicable to a wide range of cereals and pulses, these models are not suitable for all crops, such as small-seeded cereals like millets, or those that are harvested without weeds like maize. The full details of this model is described in Jones (1984, 1987).

This paper presents the R package CropPro, which provides, for the first time, openly accessible tools to conduct the same types of analysis as Jones (1984, 1987) and Charles (1998), as well as open access to the dataset behind the models, allowing anyone to use this method (ESM 1). CropPro enables the classification and comparison of archaeobotanical samples against the ethnographic data from Amorgos (ESM 1, Jones 1990). Three methods can be employed: triangular plotting, which compares the proportions of grains, rachis nodes and weed seeds, in order to gain insight into the processing of free-threshing cereals (see Jones 1990); a discriminant analysis that utilises the attributes of weed seeds to identify the products and by-products of cereal and pulse crop-processing (see Jones 1984, 1987); and another application of discriminant analysis, which again employs the attributes of wild/weed seeds, to assess the relevance of crop-processing versus alternative taphonomic pathways such as dung burning (see Charles 1998).

## Background

Using the ethnographic data collected on Amorgos, Jones (1984, 1987) introduced a method for characterising products and by-products of the crop processing sequence from which archaeobotanical material is derived. Data from the processing of cereals and pulses (bread and macaroni wheat, six rowed hulled barley, oat, pea, lentil, common vetch, and grass pea) has been used to create predictive models to classify suitable archaeobotanical samples (e.g. those with a sufficient number of items). Three by-products and one product were selected for sampling because these would most likely be kept for later use, and so potentially recovered archaeologically. Discriminant analysis, a multivariate statistical technique and form of machine learning, was used to create a model based on key physical characteristics of the weed seeds accompanying the crop during processing. This model was subsequently used to classify the archaeobotanical samples. The three characteristics of the weed seeds used are: (1) the size of the seeds relative to the fine sieve mesh used to separate small weed seeds from cereal grain, (2) the tendency of the seeds to remain in seed heads, spikes or clusters after threshing and (3) aerodynamic properties (see Table 1) (Jones 1984). By utilizing these characteristics instead of specific species to distinguish crop-processing stages, the method can be widely applied both temporally and geographically. By using Jones’s (1984, 1987) method, archaeobotanical samples can be classed (with varying degrees of probability) as one of the four sampled (by-)products: winnowing by-product, coarse sieve by-product, fine sieve by-product and fine sieve product.

**Table 1:** Weed seed characteristics based on size, tendency to remain in heads and aerodynamics, and the abbreviations used for the combinations of the weed seed characteristics

Attribute	Definitions	Combinations of characteristics
Big vs. small	Based on the likelihood of passing through the fine mesh sieve used at a late stage of processing (while retaining most of the grain)	BHH – big, headed and heavy
Headed vs. free	Based on the tendency to remain in seed heads, spikes or clusters after threshing, and so the likelihood of being retained by the coarse mesh sieve used at an early stage of processing (while allowing most of the grain to pass through)	BFH – big, free and heavy
Heavy vs. light	Aerodynamics properties relating to behaviour during winnowing: weight and attachments which aid aerodynamics such as wings or pappi	SHH – small, headed and heavy
		SHL – small, headed and light
		SFH – small, free and heavy
		SFL – small, free and light

Charles (1998) developed a modified version of Jones’s discriminant analysis method to explore the impact of alternative depositional pathways, specifically dung burning, on the archaeobotanical ‘weed’ flora, with the aim of investigating whether or not an archaeobotanical assemblage matched an alternative source more closely than those of crop-processing. While the Jones (1984) discriminant analysis method used a discriminant model that best separated four ethnographic crop processing groups based on weed seed attributes, Charles (1998) introduced archaeobotanical samples during the model’s construction (the discrimination phase), making five groups instead of four, encompassing the four crop processing groups plus an archaeological group. During the classification stage, the archaeobotanical samples were re-entered and classified as one of these five groups. This re-classification process helps determine whether the archaeobotanical samples exhibit greater similarity to the archaeological group or to the crop processing groups. By considering alternative pathways, this approach recognises that archaeobotanical material may in fact have entered the archaeological record from sources other than crop-processing. The full details of this model are described in Charles 1998).

Jones (1990) presented an additional, complementary method for understanding crop processing, based on a method used to distinguish between grain producer and consumer sites in the Thames Valley (Jones 1985). This method compares the proportions of grains, rachis nodes and weed seeds in archaeobotanical data with those in the Amorgos ethnographic data. This method utilises distinct proportions associated with different ethnographic processing stages, permitting an investigation of how closely archaeobotanical proportions align with the four crop processing (by-)products. However, because this method incorporates cereal plant parts (grain and chaff) – which are separated at different stages of crop processing depending on the type of cereal (glume wheat or free threshing cereal) – this method (based on ethnographic samples of free threshing wheat and barley) is only applicable to archaeobotanical free-threshing cereals.

### Crop processing and discriminant analysis

Two of the methods available within CropPro use discriminant analysis. Discriminant analysis uses data supplied (the ethnographic data) to build a predictive model of group membership. The method creates discriminant functions, which best discriminate between groups of the provided predictor data (the ethnographic data). As the membership of the ethnographic data is known – i.e. which crop processing stages it is from – the model builds discriminant functions which discriminate between the attributes of these groups (the seed attributes) to find the best separation. The discriminant functions produced can then be used to predict which group unknown cases (the archaeobotanical data) best fit in (one of the four crop processing stages) to varying degrees of probability.

The Charles (1998) method uses discriminant analysis in a slightly different way. Instead of using just the ethnographic data to build the model and the discriminant functions, it includes the archaeobotanical samples to build the predictive model. So, when the archaeobotanical samples are the classified against the model, there are five classes into which the archaeobotanical samples could be classified. The archaeobotanical samples, while in the model, will not necessarily be reclassified into the archaeological group. This is because the model analyses how similar the samples are to all five classes, not just the archaeological group. The method provides an understanding of how similar or different the archaeobotanical samples’ seed attributes are to material resulting from crop processing, unlike the Jones method, which selects the closest match from among the four crop processing groups in the model.

The Charles (1998) method uses the archaeobotanical samples as the extra group due to limited availability of required data on the attributes of weed seeds found in non-crop processing activities (e.g. dung-burning). Further ethnographic or experimental work could provide data to fill this gap, but it should be remembered that the objective at this stage is to show whether the archaeobotanical material is similar to that generated by crop processing or not, rather than classify the material as the remains of dung burning or other specific activities. Additional steps are required to understand whether for example dung-burning contributed to an assemblage (for full details see Charles 1998).

### The R package CropPro

The CropPro package is a collection of functions that can be used to organise and transform raw archaeobotanical data, to construct triplots in comparison with the Jones (1990) proportions of grains torachis nodes toweed seeds, to conduct discriminant analysis to compare archaeobotanical data against the Amorgos ethnographic data (ESM 1) and to plot the archaeobotanical discriminant scores against the ethnographic data’s discriminant scores. The functions can be divided into three groups: data organisation, classification and visualisation.

### Data organisation

The function crop.dataorg transforms raw archaeobotanical data into the required format for the discriminant analysis based CropPro functions. crop.dataorg calculates the square root of the percentage of weed seeds in each sample and then sums them for the different weed seed attribute categories. crop.dataorg produces a dataset with columns for each of the six combined weed-seed attributes and samples as the rows. An example of this is provided below (see the section ‘Discriminant analysis’).

### Classification

There are two discriminant analysis functions:

1. **LDAcrop.pro** follows the Jones (1984) method and uses the ethnographic data to construct a discriminant model, against which the archaeobotanical samples are classified as one of the four groups (winnowing by-product, coarse sieve by-product, fine sieve by-product or fine sieve product), classifying the entered archaeobotanical samples and providing the probabilities of their occurrence in each one of the four groups and their linear discriminant scores.
2. **LDAcrop.plus** follows the Charles (1998) method, using the ethnographic data plus the archaeobotanical samples to construct the model. The archaeobotanical samples are then reclassified against that model; samples can be classified as one of five different groups (archaeological or the four listed above).

### Visualisation

The results of the classification functions can be plotted as either a two- or three-dimensional plot. crop.plot2D produces a two-dimensional plot from the output of LDAcrop.pro, in which the user can select which discriminant function will be shown on which axes. crop.plus_plot2D works in the same way as crop.plot2D, but plots the output of LDAcrop.pro. crop.plot3D and crop.plus_plot3D using the outputs of the two LDA functions to plot the first three discriminant functions as an interactive three-dimensional plot^1^. Another visualisation function is crop.triplot, which plots data from the proportions of grains torachis nodes toweed seeds within samples and compares them to the ethnographic data’s proportions. An example of this is provided below (see the section ‘Triplots’).

### Use of the CropPro package

**Fig. 1:**
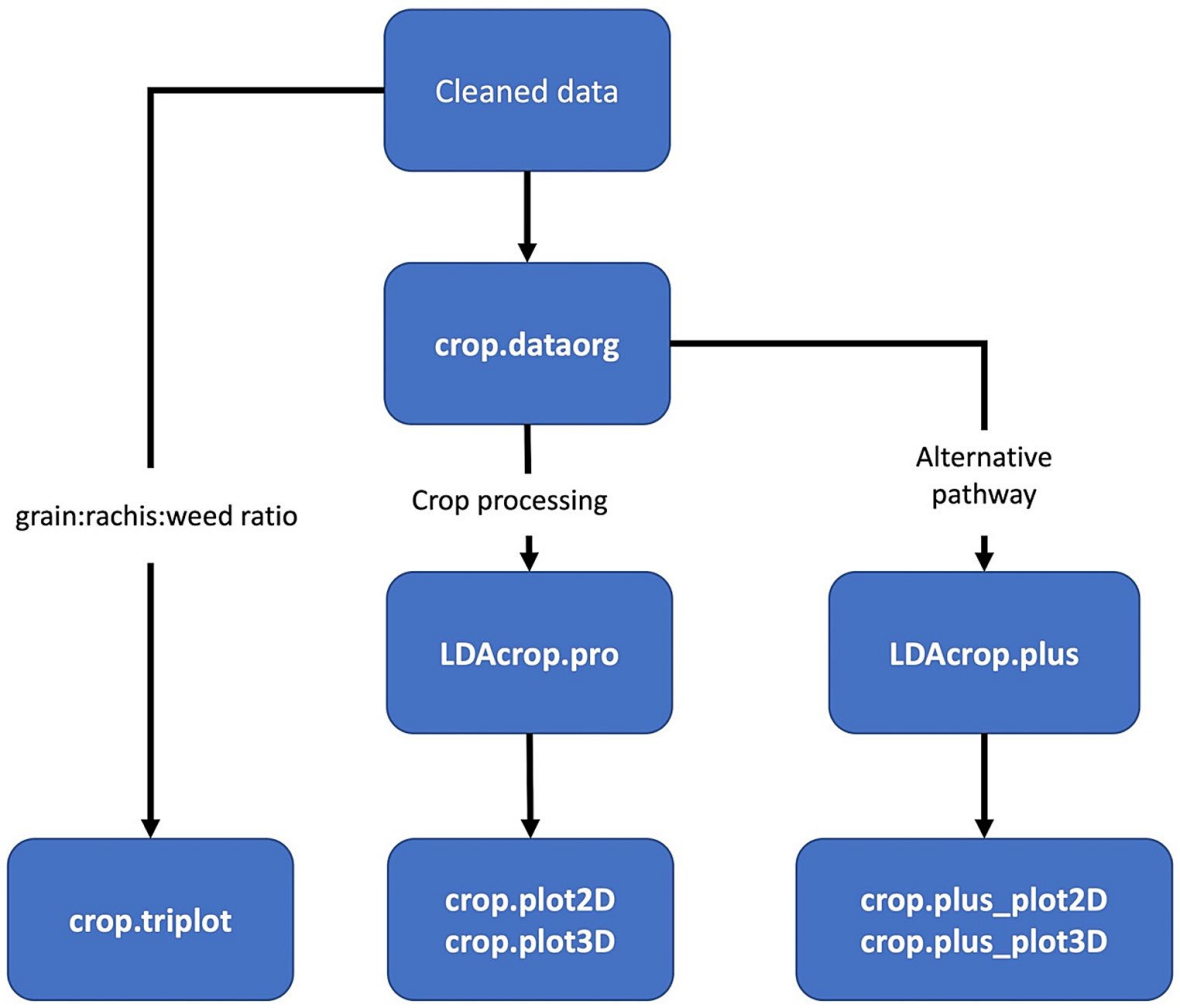
Flow diagram of the main processes and functions of the CropPro package

The CropPro package offers a range of functions that can be used in a variety of workflows. The workflow followed below is the best order for the example datasets provided; however, it should be noted that workflow will vary depending on the assemblage analysed and the research questions posed. It is recommended to use the functions in an exploratory way to investigate the archaeobotanical assemblage, trying out alternative classifications and thresholds to better understand the implications. In the examples below, the package is applied to a temperate European dataset (Stafford) and to a semi-arid south-west Asian dataset (Tell Brak). Figure 1 provides a simplified flow diagram outlining the main steps required to conduct the three different analyses.

Users of the package should have a comprehensive understanding of their dataset, including the proportions of items within each sample, the dominance of specific crops and the research questions being addressed. For methods based on weed/wild seed attributes alone, we recommend an absolute minimum of 10 seeds per sample, although analyses based on larger numbers would be much more reliable. A minimum of 10 weed seeds per sample is suggested as a compromise between reliability (the lower the minimum number per sample, the less reliable the classification of the sample) and the inclusion of samples in the analysis (the higher the minimum number per sample, the fewer the number of samples included), which can result in an unrepresentative assemblage of samples. No minimum number of weed seeds is required for inclusion in the triplot method, where the percentages of weed seeds, grains and rachis nodes are used to create the plot.

The quality of the information obtained from the analyses can vary according to context, with mixed crop types from secondary or tertiary deposits being more challenging to interpret, given that they likely derive from multiple events. While not essential, an understanding of patterns based on context type, density and crop type is helpful. The authors have found correspondence analysis to be informative in ascertaining patterns that may aid in understanding the taphonomic pathway of the samples. An example demonstrating this process is described in Bogaard et al. (2021).

The package can be downloaded into R from GitHub^2^ using the devtools package by Wickham et al. (2022). The package CropPro can be manually downloaded from the CropPro GitHub account or download it within R using the devtools package’s function install_github (see ESM 2: code line 6).

### Stafford

The early medieval site of Stafford was occupied from the late 7th century onwards, and the archaeobotanical samples used here date from the 9th to 16th centuries. Excavations at a number of locations around the town produced a quantity of archaeobotanical remains. The raw data are derived from the original archaeobotanical analyses conducted by Moffett (1987) and Druce (2014) and can be found in McKerracher et al. (2023). The phasing used in this paper was devised by the FeedSax project (Hamerow et al. 2020). The R script created to analyse the dataset for this paper is provided and specific code lines referred to throughout the demonstration of the package (ESM 2). The dataset used here has been simplified for ease of demonstration (ESM 3): analysis of the complete dataset without omissions is available in McKerracher et al. (2023). The Stafford dataset consists predominantly of free-threshing cereals, with glume wheat forming a negligible proportion of the assemblage, making it comparable to the ethnographic data.

To use the CropPro package, the dataset was cleaned, with tentatively identified specimens (i.e., cf. identifications) re-assigned to positively identified categories, or demoted to wider classification groups (genus or family groups). Specimens that were not seeds or rachis nodes were removed, for example culm, calyx tips and pod fragments. Non-arable items were removed, including any edible species such as fruits and nut species (e.g. for the Stafford data *Prunus* fruit stones were removed). Understanding what is non-arable can be an iterative process, involving the inclusion/exclusion of species and examination of the impact, or facilitated through the use of correspondence analysis. The weed seed species were classified using Jones’s categories (see Table 1 for categories, see below for more detail). Any weed seed, which could not be classified, was left blank (see ESM 3, column “Codes”).

#### Triplots

To investigate crop processing using the proportions of grains torachis nodes toweed seeds, the dataset was further cleaned: any pulse and flax items and the single spelt grain were removed and only the free-threshing cereal used. From this simplified and cleaned dataset the total grain, rachis nodes and weed seeds per sample were calculated, with only samples that contained at least 30 items included (sample 1174 was removed, ESM 2: code lines 18–20). The cut-off for total number of items per sample is assemblage-dependent and should be modified given the richness of the assemblage. If the number of samples in the assemblage is large, then the minimum number of items per sample could be raised to include only the most statistically reliable samples but, if the number of samples is small, reducing the numbers further may result in an unrepresentative assemblage of samples. To use the function crop.triplot, the data needed to be orientated with samples in rows and the three categories in columns (Table 2, ESM 2: code lines 23–24). It is also possible to do the above data manipulation outside R and to import a dataset that has samples in rows and three columns with the total numbers of grains, rachis nodes and weed seeds (Table 2).

**Table 2:** A portion of the input data for crop.triplot showing the required format

Sample	Grain	Rachis	Weeds
461	23,173	2,300	9,760
462	239	56	73
463	102	26	27
464	264	360	19
465	245	276	100
466	2,060	437	3,567
467	1,327	153	2,228

**Fig. 2:**
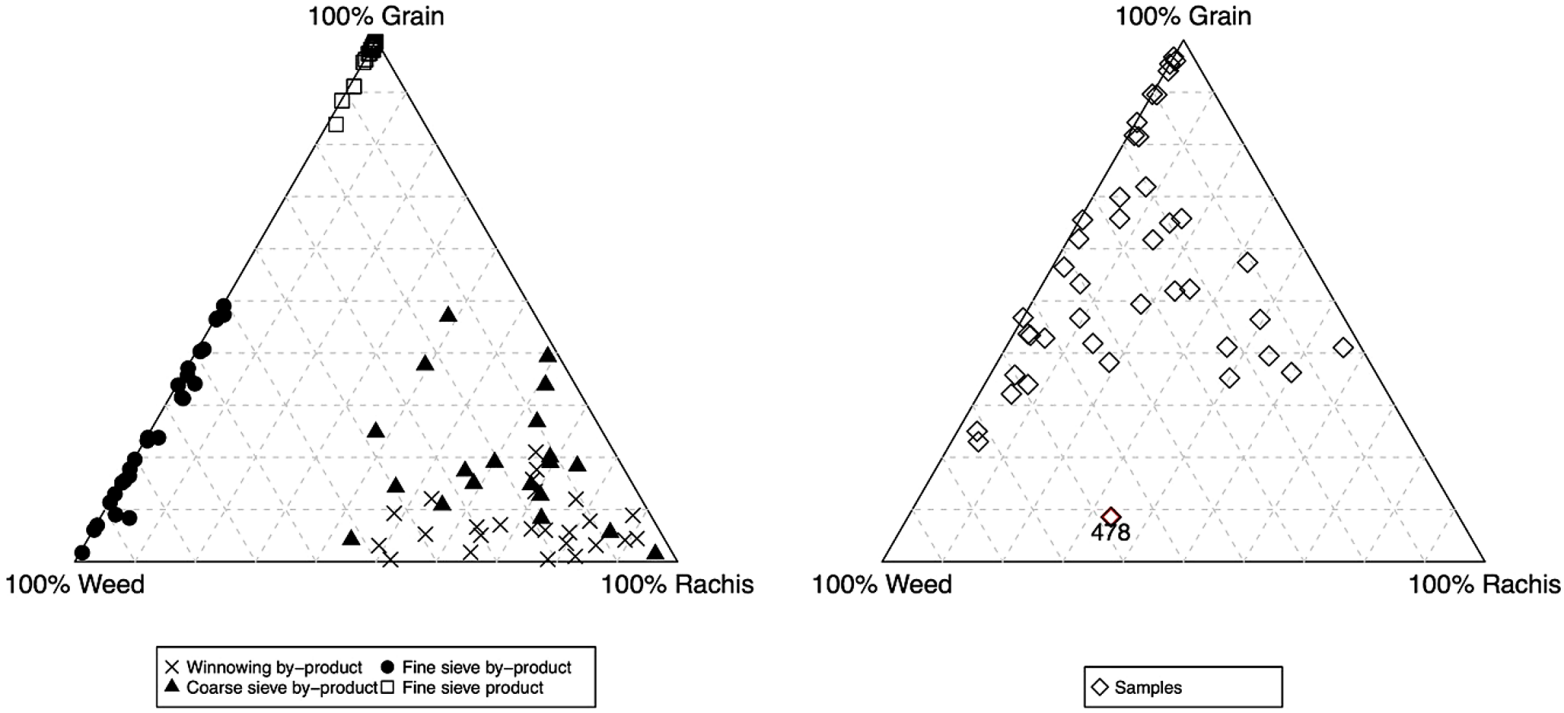
The plots produced using crop.triplot showing the ethnographic data (left) and the Stafford data (right). Sample 478 is highlighted

The function crop.triplot plots the inputted data, as well as the proportions of the ethnographic data; these two graphs are displayed side-by-side in the outputted graph (Fig. 2). crop.triplot has multiple defaults, allowing the symbol’s colour/outline, the symbol’s infill colour and the symbol’s shape to be modified for both the ethnographic and archaeobotanical data. Specific samples can also be labelled and/or highlighted based on row number. When the Stafford data are plotted using crop.triplot the result shows that a high proportion of samples fall in the cleaned products region of the graph, while the other samples appear to be a mixture of multiple crop processing stages (ESM 2: code line 25, Fig. 2). A small number of samples have proportions similar to coarse sieve by-product and fine sieve by-product. One sample falls outside the main grouping, with a low percentage of grains compared to weed seeds and rachis nodes. Using crop.triplot’s argument “sample”, the sample 478 can be highlighted and labelled (ESM 2: code line 26, Fig. 2).

#### Discriminant analysis

Discriminant analysis was used to compare the attributes of the weed seeds of the Stafford assemblage to the ethnographic data. The discriminant analysis provided an understanding of how similar the Stafford data were to each of the four crop processing groups. Data cleaning was conducted to remove any grain and rachis entries used in the previous triplot analysis, leaving only weed seeds. To conduct the discriminant analysis, the weed taxa needed to be classified based on their seed size, tendency to remain in heads and aerodynamic properties. Multiple methods can be used to classify the species: previously published data on relevant species can be used as well as personal measurements and experimental data. The classification of the Stafford species is shown in ESM 4, along with additional species relevant to archaeobotanical assemblages. Furthermore, the user needs to judge what delineates small vs. big, light vs. heavy, headed vs. free for their assemblage, as this may vary (e.g. 1.5 and 2 mm cut-offs for small vs. big could be compared). For the Stafford data any item which could not be classified was removed and only samples which had 10 or more classifiable items were included in the analysis, resulting in 41 usable samples (ESM 2: code lines 31–40). Such a cut-off is, again, assemblage-dependent; a minimum of 10 items per sample was set for the Stafford dataset. It is also possible at this stage, to enter a spreadsheet into R, in which all the above manipulations have been conducted outside R.

**Fig. 3:**
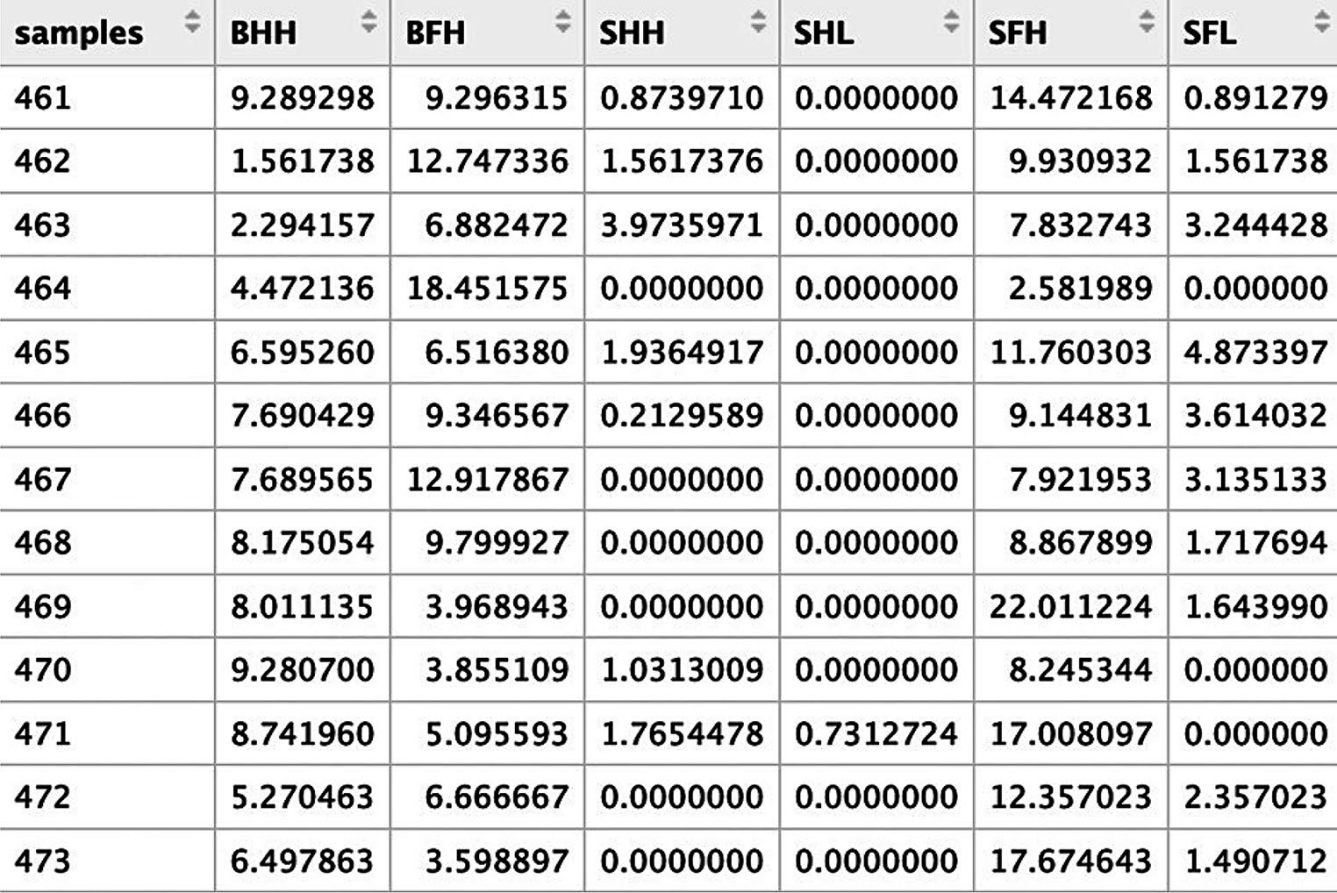
A portion of the output of crop.dataorg for the Stafford data

The finalised, cleaned and labelled dataset was transformed and organised using the function crop.dataorg, which conducts a square root transformation on the data (see Jones 1984, p 49). crop.dataorg requires information regarding which column contains the seed attribute codes and which column contains the first sample (ESM 2: code line 43). crop.dataorg produces a table of the summed, transformed values of the different species classified as either BHH, BFH, SHH, SHL, SFH or SFL, for each sample (Fig. 3). The crop.dataorg output is also in the correct orientation for discriminant analysis.

**Fig. 4:**
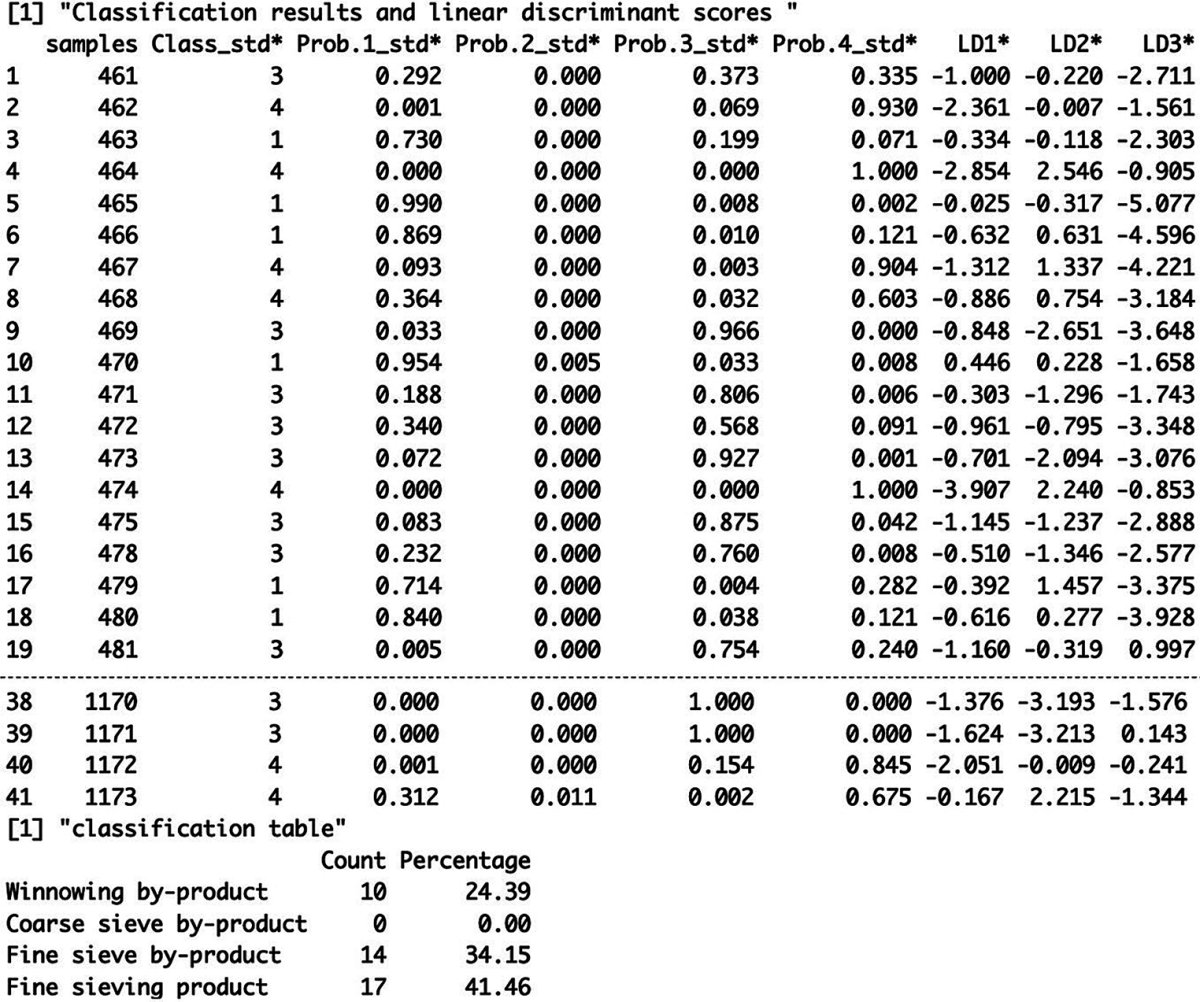
A portion of the R console output of LDAcrop.pro showing the results table and the classification table of the Stafford data

LDAcrop.pro is one of the two linear discriminant functions in the CropPro package and it classifies the entered archaeobotanical data against a discriminant model constructed using the ethnographic data. LDAcrop.pro is simple to use, only requiring the output of crop.dataorg to be entered (ESM 2: code line 45). The results of LDAcrop.pro are printed in the console and show the classification of the samples, the probability of the sample being classified as group 1, 2, 3 or 4 and the linear discriminant scores for function 1, 2 and 3 (Fig. 4). A classification table is also produced which shows the numbers and percentages of samples classified as winnowing by-product (group 1), coarse sieve by-product (group 2), fine sieve by-product (group 3) or fine sieve product (group 4) (Fig. 4).

The results show that 41% of the Stafford samples are classed as fine sieve product, with no samples classified as coarse sieve by-product (Fig. 4, “classification table”). When interpreting sample classification, examination of the probability columns provides an understanding of how well the samples fit in their assigned group – that is, how similar the samples are to that processing group as opposed to the other groups. A probability of 1 (100%) means that that sample strongly resembles that group compared to the other groups; it does not mean it has the same composition, just that it is much more dissimilar to the other groups. Examination of the classification probabilities (columns Prob.1_std*, Prob.2_std*, Prob.3_std* and Prob.4_std*, Fig. 4) shows that the samples classified as winnowing by-products (Class 1) all have a greater than 70% probability. The probabilities of the samples classified as fine sieve by-products show that sample 461 has a 37% probability of belonging in that group but that it also has a 29% chance of being a winnowing by-product and a 34% chance of being a fine sieve product. Furthermore, among the samples classified as fine sieve products, six have less than 70% chance of belonging in that group. Such results indicate that some of the samples conform closely to one or other of the four processing (by-)products but other samples do not, potentially indicating a mixture of (by-)products, the inclusion of material from non-crop-processing activity or the most likely interpretation, given the greater probability (second choice) of fine sieved by-products, an intermediate product of unsieved grain.

The results of LDAcrop.pro, when saved as an object, provide additional information (ESM 5). The columns denoted by an asterisk are those that are used throughout this analysis and in subsequent functions. The MASS package that is used within the LDAcrop.pro function to conduct the linear discriminant analysis provides standardised and unstandardised data that are shown in the additional columns (see the CropPro help document; Stroud et al. (2023), or Venables and Ripley (2002) for full details). The unstandardised linear discriminant scores (LD1*, LD2* etc.) are used in the plotting functions below. Furthermore, the standardised probability (Prob.1_std* etc.) and classifications (Class_std*) should be used when assessing the results.

**Fig. 5:**
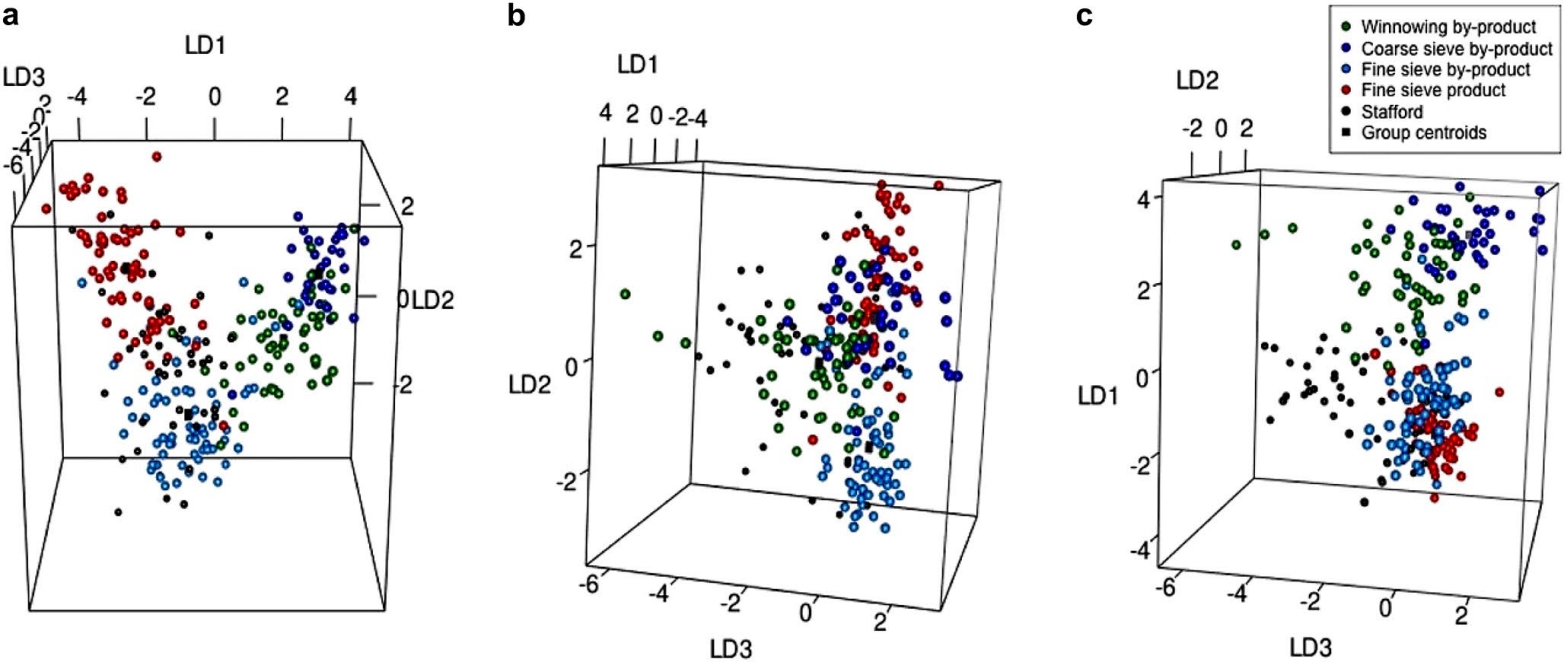
Static images of the interactive plot produced by the function crop.plot3D from the discriminant analysis of the Stafford data using LDA.croppro, **a**, a static image of the first and second axes (Linear discriminant function (LD) 1 and 2), **b**, the second and third axes (Linear discriminant function (LD) 2 and 3), and **c**, the third and first axes (Linear discriminant function (LD) 1 and 3)

Plotting the linear discriminant scores also illustrates how well the samples conform to the ethnographic groups. CropPro has two plotting options for crop processing data: a two-dimensional plot and a three-dimensional plot, both using the results from LDAcrop.pro. The function crop.plot3D is a great way of visualising examining how similar the samples are to the crop processing groups, as all three discriminant functions are plotted. As the plot is interactive, it is possible to manipulate it to see where the samples fall on all three axes in comparison with the ethnographic data (Fig. 5). crop.plot3D requires the output of LDAcrop.pro, and will extract the three linear discriminant functions to create the plot. The colour of the entered archaeobotanical data as well as the ethnographic data can be changed with the arguments of *col* and *gcol* respectively. Finally, the argument *site* allows users to change the label of the archaeobotanical data in the legend. While this paper has images of crop.plot3D as examples, it should be noted the crop.plot2D can provide a 2D version of the differing axes for publication; crop.plot3D can provide images but requires the user to originate the graph to the correct angle and can be harder to understand visually in a static form.

Plotting the Stafford data using crop.plot3D (ESM 2: code line 47) provides an interactive graph showing the data in relation to the ethnographic data: it shows that the samples plot near the fine sieve product and by-product groups, on the first two discriminant functions (Fig. 5a). However, when the graph is rotated to display discriminant function 2 and 3, the samples extend out on the third discriminant function axis, similar to the winnowing by-products (hence the reason 10 samples were classified as winnowing by-products) (Fig. 5b). Rotating the graph again to show discriminant function 1 and 3, the archaeobotanical samples classified as winnowing do not directly plot over the ethnographic data; instead some fall outside the distribution of the ethnographic data (Fig. 5c). It is most likely that those samples are a mixture of processing stages.

**Fig. 6:**
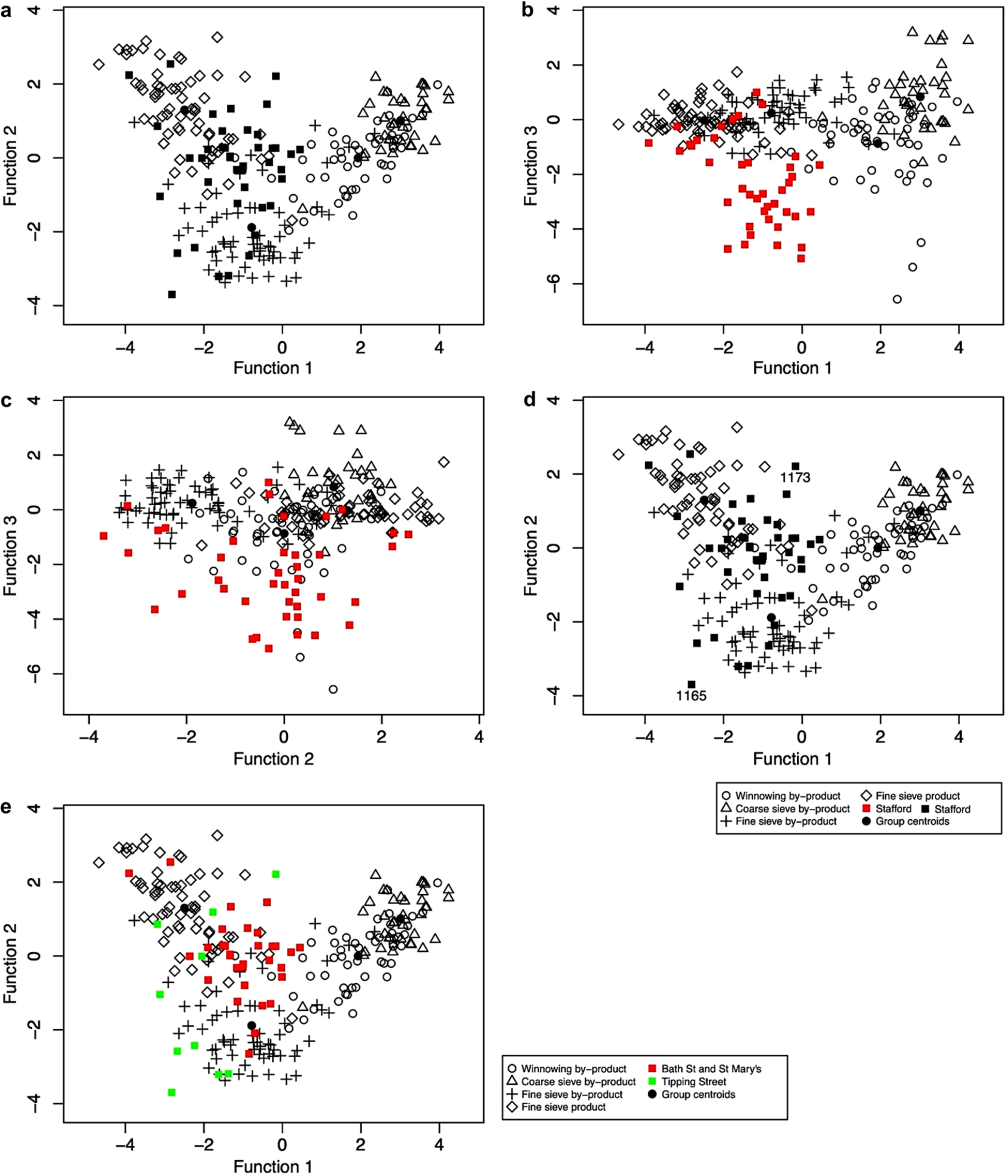
2D plots of the results of the discriminant analysis of the Stafford data using LDAcrop.pro compared against the ethnographic model; **a**, the 2D plot showing first and second discriminant function; **b**, a 2D plot of the first and third discriminant function; **c**, a 2D plot of the second and third discriminant function; **d**, a 2D plot of the first and second function with samples 1165 and 1173 labelled; **e**, a 2D plot with the samples coloured green to show the Tipping Street samples and red to show Bath St and St Mary’s samples

While crop.plot3D is a useful tool for investigating the data, it may be difficult to publish, and the function crop.plot2D provides a two-dimensional plot (Fig. 6). While it defaults to displaying the first two discriminant functions, it can be changed so that any combination of the three discriminant functions are used (see ESM 2: code lines 54–55, Fig. 6a–c). In addition, specific samples can be labelled and there are arguments which can be used to change both the symbols and their colours for both archaeobotanical samples and ethnographic data (ESM 2: code lines 73–75, Fig. 6d and e). The default is set to a black and white graph.

**Fig. 7:**
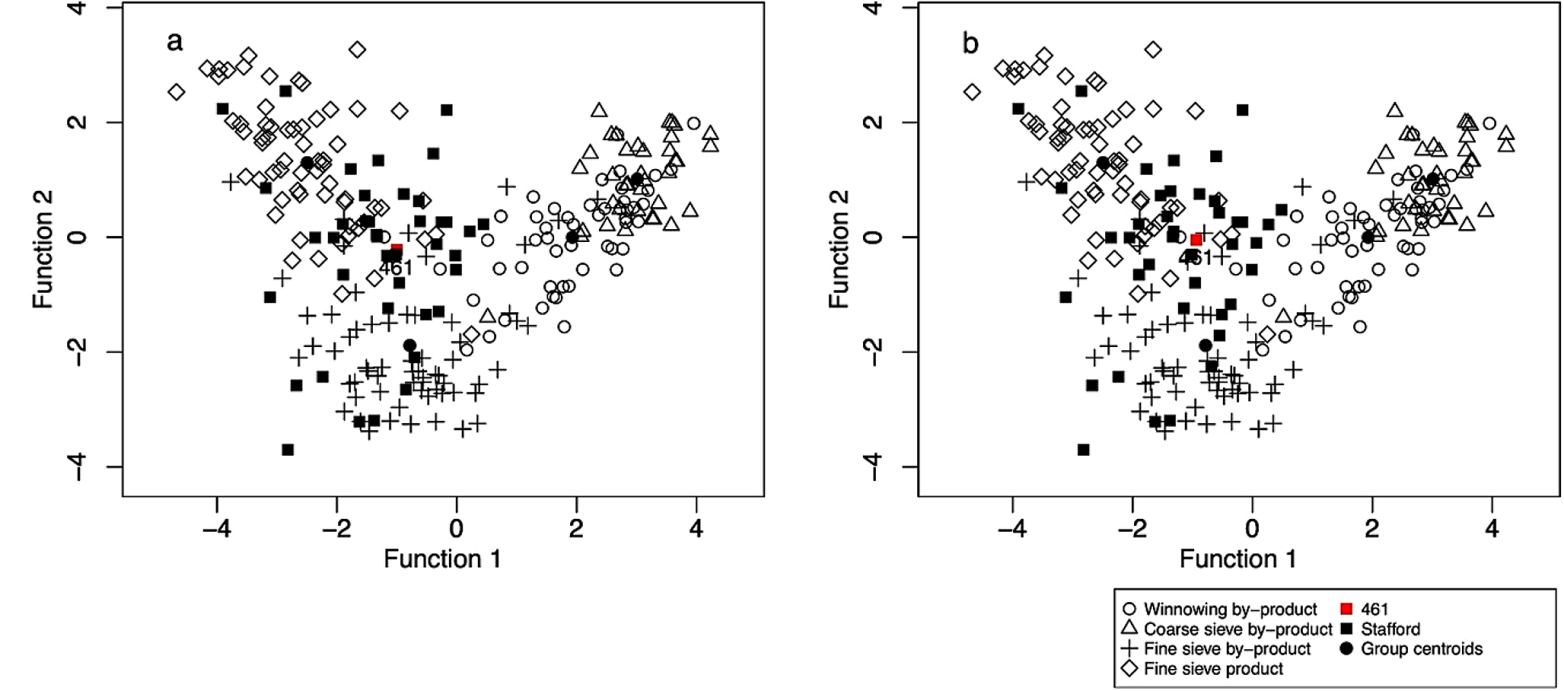
**a**, The results of the original crop processing discriminant analysis of the Stafford data with sample 461 highlighted; **b**, The results of the modified analysis of the Stafford data with the hay meadow taxa removed

The results of the Stafford analysis suggest that, while many of the samples derived from the fine sieved product, other samples do not fully align with the ethnographic data. This could be a result of a mixture of multiple processing (by-)products, or the inclusion of material from alternative sources. To investigate whether the inclusion of possible hay meadow species had an impact on the classification, species associated with hay meadows were removed (see Table 3). The analysis was then rerun, with the data organised using crop.dataorg and then analysed with LDAcrop.pro (ESM 2: code lines 85–114). There were limited changes to the results: only sample 461 changed classification, and this was the sample which had been noted previously as having a low similarity to the other groups. The limited changes highlight the insignificant impact of potential hay meadow taxa on the overall classifications. This suggests that the influence of hay meadow is limited or non-existent. Plotting the samples also shows limited differences compared to the original graph (compare Fig. 7a and b).

**Table 3:** Species removed from dataset in secondary analyses to investigate hay meadow (Stafford) and dung (Tell Brak) influence

Stafford	Reason	Tell Brak	Reason
*Eleocharis palustris/uniglumis*	Hay meadow	*Scirpus maritimus*	Dung
*Leucanthemum vulgare*		*Scirpus/Schoenoplectus*	
*Silene flos-cuculi*		*Trigonella astroites*	
		*Trigonella* indet	
		*Trigonella/Astragalus*	

### Tell Brak

To provide an example from a semi-arid location and use of the set of functions within CropPro to understand potential dung burning, the dataset from Tell Brak, a large tell site located in north-eastern Syria, was analysed. The dataset contains samples from the 3rd millennium bce phases (Late ED III, Akkadian and post-Akkadian occupation). The dataset published in Charles and Bogaard (2001) has been simplified for ease of demonstration, resulting in slight deviations from the results presented in that publication (ESM 6). The R script used for the analysis is supplied (ESM 7).

Data cleaning involved the removal of items not applicable to the analysis. Items within the dataset were classified as either free-threshing crop grains, free-threshing crop rachis, glume wheat items (grain and chaff) or weeds. Any items that fell outside such classification (e.g. dung remains, culm and wild chaff, fruits and nuts) were labelled with an “N” (ESM 6 column Cat1). This column was used in R to filter the dataset to obtain the groups necessary for the analysis (ESM 7: code line 16).

The Tell Brak dataset contains both free-threshing crops and glume wheats. Given that the ethnographic data derives from free-threshing crops, the assemblage was examined to understand the dominance of such crop types within each sample and to determine their eligibility. The samples were classified based on the proportion of crops within the samples using an 80% threshold for barley, free-threshing cereal (wheat and barley), pulse and mixed as per Charles and Bogaard (2001) (ESM 8). Barley (16 samples), lentil (2 samples) and pea (1 sample) dominate some samples, while others contained a combination of free-threshing wheat and barley items (the “free-threshing cereal” classification group, 9 samples); no sample was dominated by glume wheat items only. The remaining samples were classed as mixed (12 samples) (ESM 8).

#### Triplot

crop.triplot was used to investigate the Tell Brak data and to construct triplots showing the proportion of grains torachis nodes toweed seeds across the samples in comparison to the ethnographic data. As the ethnographic data used in the crop.triplot come only from free-threshing cereals, only free-threshing cereal dominated samples were used (those classed as “barley” or “free-threshing cereal”); all mixed and pulse samples were removed (ESM 7: code line 28). The proportions of grains, rachis nodes and weed seeds were calculated, excluding glume wheat grains and glume bases, as well as weed items which were not seeds (i.e. wild grass rachis) (ESM 7: code line 16). Any samples containing less than 30 such items were excluded (samples ST105/26, ST105/27 and ER45/13). As with the Stafford data, the Tell Brak data were orientated correctly with samples in rows and grain, rachis and weed totals in columns. The resultant cleaned and modified data were entered into crop.triplot (ESM 7: code line 32).

**Fig. 8:**
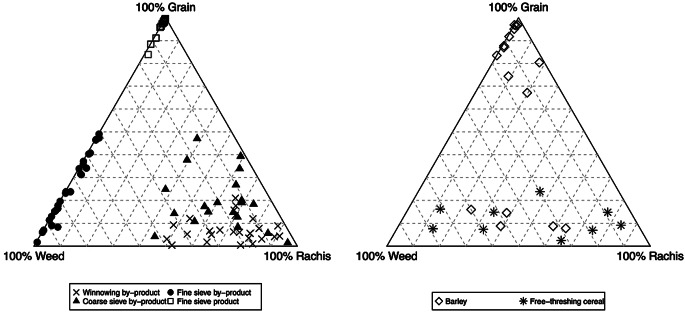
The plots produced using crop.triplot showing the ethnographic data (left) and the Tell Brak data (right)

The output of crop.triplot, coded to differentiate between the barley-dominated and free-threshing cereal-dominated samples, shows that the barley samples predominantly plot in the region of cleaned product due to the dominance of grain within the samples (Fig. 8). The low-grain samples, predominately the “free-threshing cereal group”, plot towards the rachis/weed side of the graph, the region in which the ethnographic samples from winnowing/coarse sieve by-products occur (Fig. 8, ESM 7: code line 38).

#### Discriminant analysis

Further investigation of the crop processing stages represented in the Tell Brak data was conducted using discriminant analysis. The dataset was cleaned to remove any crop or collected species. The remaining weed taxa were classified based on their size, tendency to remain in heads and aerodynamics (see ESM 6, column “codes”). Any specimen that could not be classified – either due to lack of information, or because it was not identified to a species or genus type with uniform attributes – were removed. For the Tell Brak assemblage the minimum number of items per sample threshold was set at 20 to provide a selection of samples, which were more representative of the overall assemblage. As explained above it is recommended that users test different variations for all decisions made (classifications, and number of items per sample) to see whether the results change for their assemblage. Such iterative use is not shown below due to limited space.

**Fig. 9:**
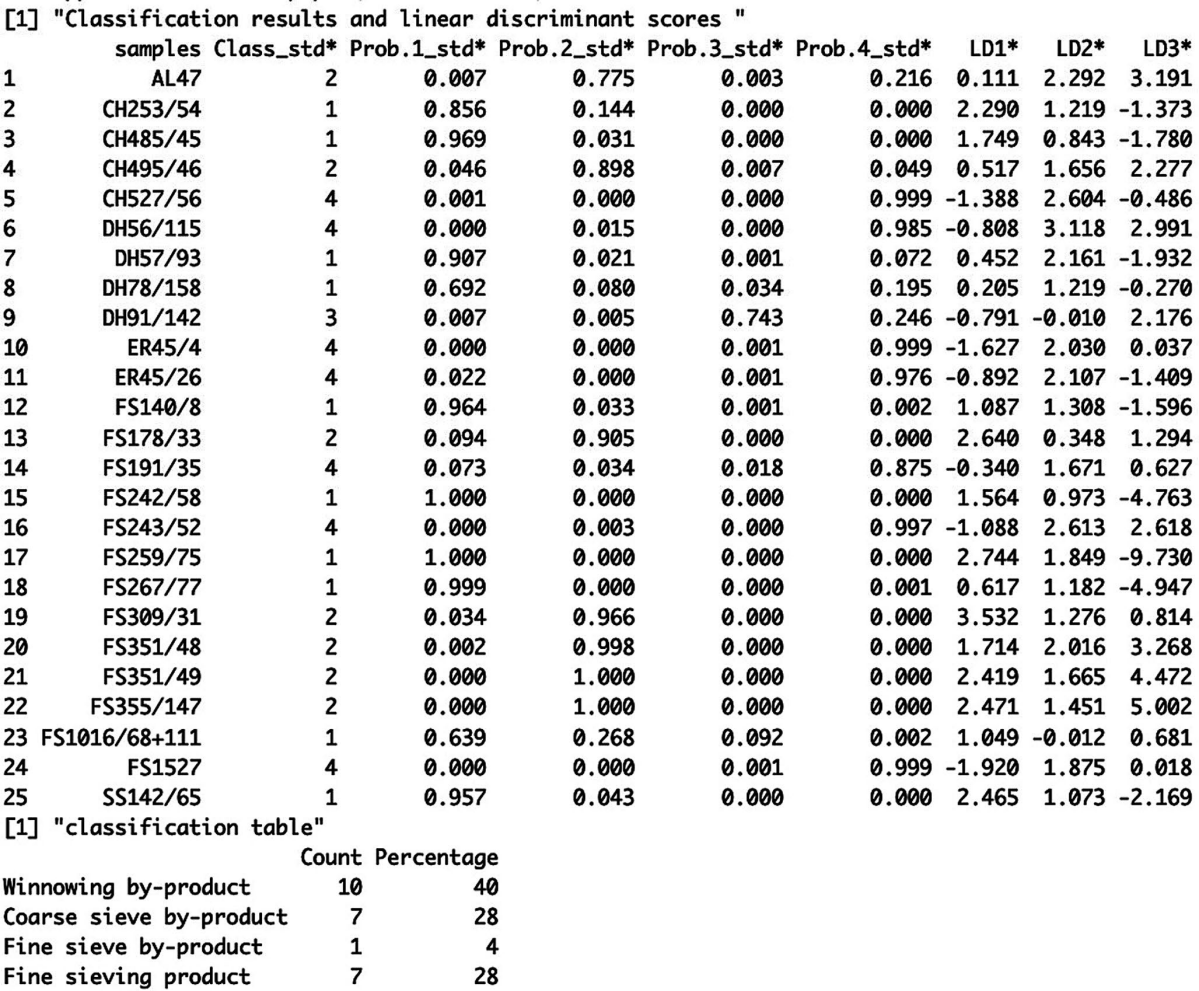
A portion of the R console output of LDAcrop.pro showing the results table and the classification table of the Tell Brak data

To arrange the cleaned data into the correct format as well as conduct a square root transformation, the function crop.dataorg was used (ESM 7: code line 58). The output was then analysed using LDAcrop.pro (ESM 7: code line 60), with the results indicating a relatively even distribution of samples between winnowing by-products, coarse sieve by-product and fine sieve products (30–40%) (Fig. 9). Classification probabilities indicate several low values, in particular sample DH78/158 and FS1016/68 + 111(63%) likelihood of belonging to group 1, the winnowing by-product group (Fig. 9; Table 4).

**Table 4:** The LDA classification of the Tell Brak samples, which group they are in (barley-dominated, free-threshing cereal-dominated (Ft) and mixed composition), and their probability of being in class 1, 2, 3 or 4 (winnowing by-product, coarse sieve by-product, fine sieve by-products and fine sieve product respectively)

Class	Classification	Group	Samples	Probability of being in class 1, 2, 3 or 4			
				1	2	3	4
1	Winnowing by-product	Barley	CH253/54	**0.856**	0.144	0	0
	Winnowing by-product	Barley	FS242/58	**1**	0	0	0
	Winnowing by-product	Barley	FS259/75	**1**	0	0	0
	Winnowing by-product	Ft	DH78/158*	**0.692**	0.08	0.034	0.195
	Winnowing by-product	Ft	FS1016/68 + 111*	**0.639**	0.268	0.092	0.002
	Winnowing by-product	Ft	FS140/8	**0.964**	0.033	0.001	0.002
	Winnowing by-product	Ft	SS142/65	**0.957**	0.043	0	0
	Winnowing by-product	Mixed	CH485/45	**0.969**	0.031	0.000	0
	Winnowing by-product	Mixed	DH57/93	**0.907**	0.021	0.001	0.072
	Winnowing by-product	Mixed	FS267/77	**0.999**	0	0	0.001
2	Coarse sieve by-product	Barley	CH495/46	0.046	**0.898**	0.007	0.049
	Coarse sieve by-product	Barley	FS355/147	0	**1**	0	0
	Coarse sieve by-product	Ft	FS178/33	0.094	**0.905**	0	0
	Coarse sieve by-product	Mixed	AL47	0.007	**0.775**	0.003	0.216
	Coarse sieve by-product	Mixed	FS309/31	0.034	**0.966**	0	0
	Coarse sieve by-product	Mixed	FS351/48	0.002	**0.998**	0	0
	Coarse sieve by-product	Mixed	FS351/49	0	**1**	0	0
3	Fine sieve by-product	Barley	DH91/142	0.007	0.005	**0.743**	0.246
4	Fine sieve product	Barley	ER45/26	0.022	0	0.001	**0.976**
	Fine sieve product	Barley	ER45/4	0	0	0.001	**0.999**
	Fine sieve product	Barley	FS1527	0	0	0.001	**0.999**
	Fine sieve product	Ft	CH527/56	0.001	0	0	**0.999**
	Fine sieve product	Ft	FS191/35	0.073	0.034	0.018	**0.875**
	Fine sieve product	Mixed	DH56/115	0	0.015	0	**0.985**
	Fine sieve product	Mixed	FS243/52	0	0.003	0	**0.997**

* denotes samples with low probabilities for their classification group

Overall, the free-threshing cereal and barley samples are predominantly classified as winnowing by-product or fine sieve product, agreeing with the grains torachis nodes toweed seeds proportions, which indicate that samples are either fine sieve products or fall into the winnowing/coarse sieve by-product area of the triplot.

**Fig. 10:**
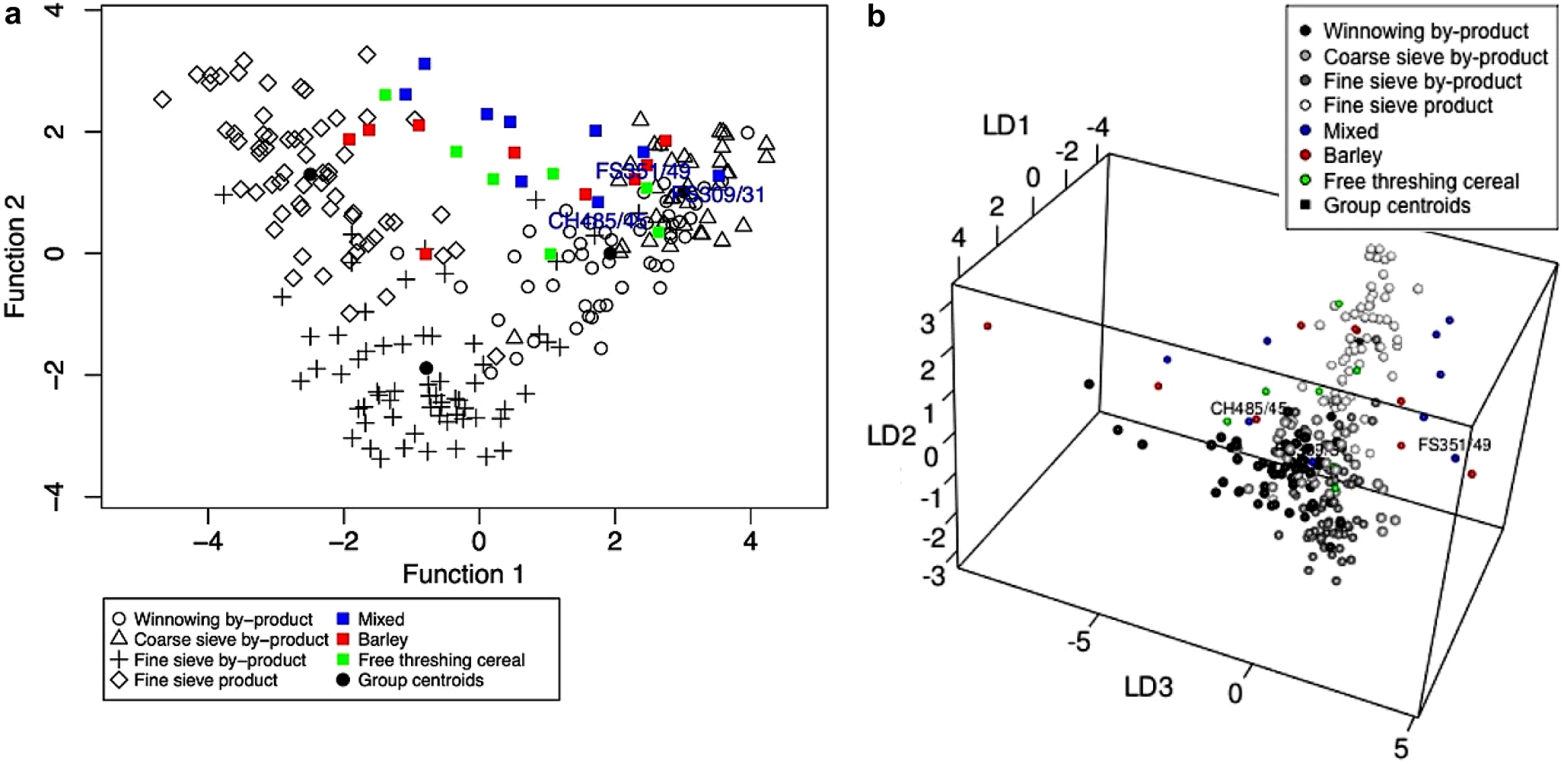
**a**, a plot of the Tell Brak discriminant analysis results created using crop.plot2D, with the samples colour coded based on sample composition and **b**, a plot of the Tell Brak discriminant analysis using crop.plot3D with the samples colour coded based on sample composition and the plot rotated to show the 2nd and 3rd axes

Plotting the results using crop.plot2D function, where the samples are colour-coded based on their classification group (barley, mixed and free-threshing cereals), highlights the location of the samples (ESM 7: code lines 69–74) (Fig. 10a). The mixed samples plot outside the ethnographic groups in the upper centre space, highlighting their mixed nature. The exceptions to this are samples FS309 and FS351/49, which plot within the coarse sieve by-product group, and CH485/45, which plots within the winnowing by-product group (Fig. 10a). Using crop.plot3D with these three samples labelled, it can be seen that while FS309 and CH485 conform to their groups on the three-discriminant axis, FS351 plots slightly outside the coarse sieve by-product group on the third axis (Fig. 10b) (ESM 7: code lines 81–82). Rotating the crop.plot3D also shows that on the third axis the majority of mixed samples do not overlap with the processing groups.

**Fig. 11:**
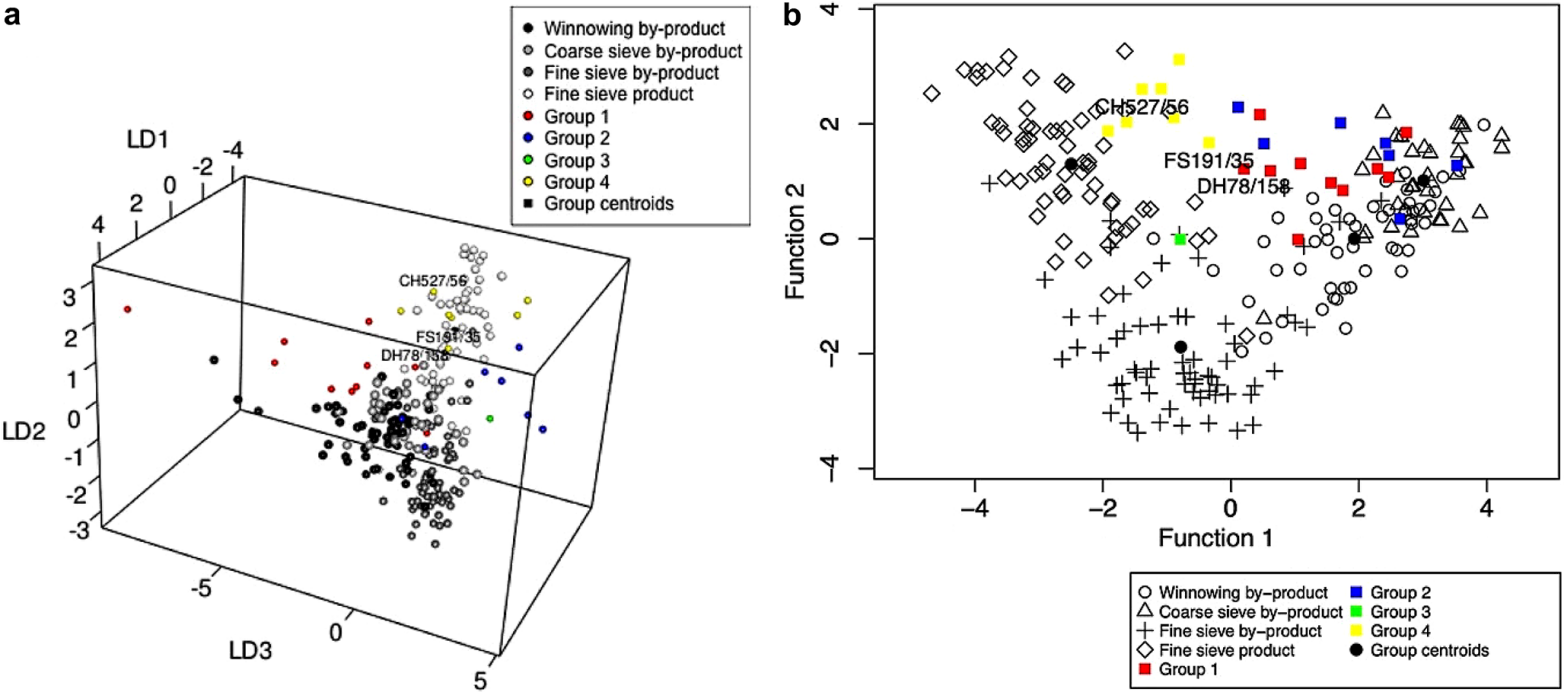
**a**, a plot of the Tell Brak data created using crop.plot3D with the samples coloured based on LDA classification and the plot rotated to show the 2nd and 3rd axes; **b**, a plot of the Tell Brak data created using crop.plot2D with the samples coloured based on LDA classification

When the samples are colour-coded based on their LDA classification using crop.plot3D it can been observed how close the samples are to the centroids of the crop processing groups and how they behave on the third axis: winnowing samples (group 1) pull out along the negative side, coarse sieve samples (group 2) on the positive side along axis 3 (Fig. 11a). There are two free-threshing cereal samples which are classified as fine sieve product (CH527/56 and FS191/35); FS191/35 plots on the periphery of the fine sieve product group while CH527/56 plots towards the middle (Fig. 11b). Examination of the other components within the samples reveals a high proportion of big, free and heavy *Aegilops* seeds and rachis nodes. This suggests that they are a mixture of the early stages of crop processing as well as hand-sorting residue.

It is advisable to investigate the impact particular species have had on a sample’s overall classification (see the above example for *Aegilops* seeds), the classification of species (e.g. big vs. small cut-offs) and the inclusion/exclusion of potential arable/non-arable species.

#### Use of the crop.plus functions

The CropPro package also includes a set of functions which can be used to investigate assemblages where it is uncertain that the samples are the by-products crop processing, and it is possible that other sources have contributed to the assemblage i.e. dung-burning, turf-burning etc. The crop.plus suite of functions follow Charles’s (1998) method where, unlike the linear discriminant analysis method described above (LDAcrop.pro), the ethnographic and archaeobotanical samples are used to create the model at the discrimination stage. The archaeobotanical samples are then re-classified against the created model that has five groups: the four crop processing stages and an archaeological group.

The function LDAcrop.plus discriminates the archaeobotanical samples and four crop processing groups, creating a model that is assemblage-dependent. The use of LDAcrop.plus is very similar to LDAcrop.pro: the output of crop.dataorg can be entered into LDAcrop.plus with no modification, making is easy to conduct both LDAcrop.pro and LDAcrop.plus from the same output. The output of LDAcrop.plus is also similar to that of LDAcrop.pro, with the classification of the samples, probabilities and discriminant scores shown in the console, along with a classification table showing the percentages of samples classified as archaeological, or one of the four crop processing stages.

**Fig. 12:**
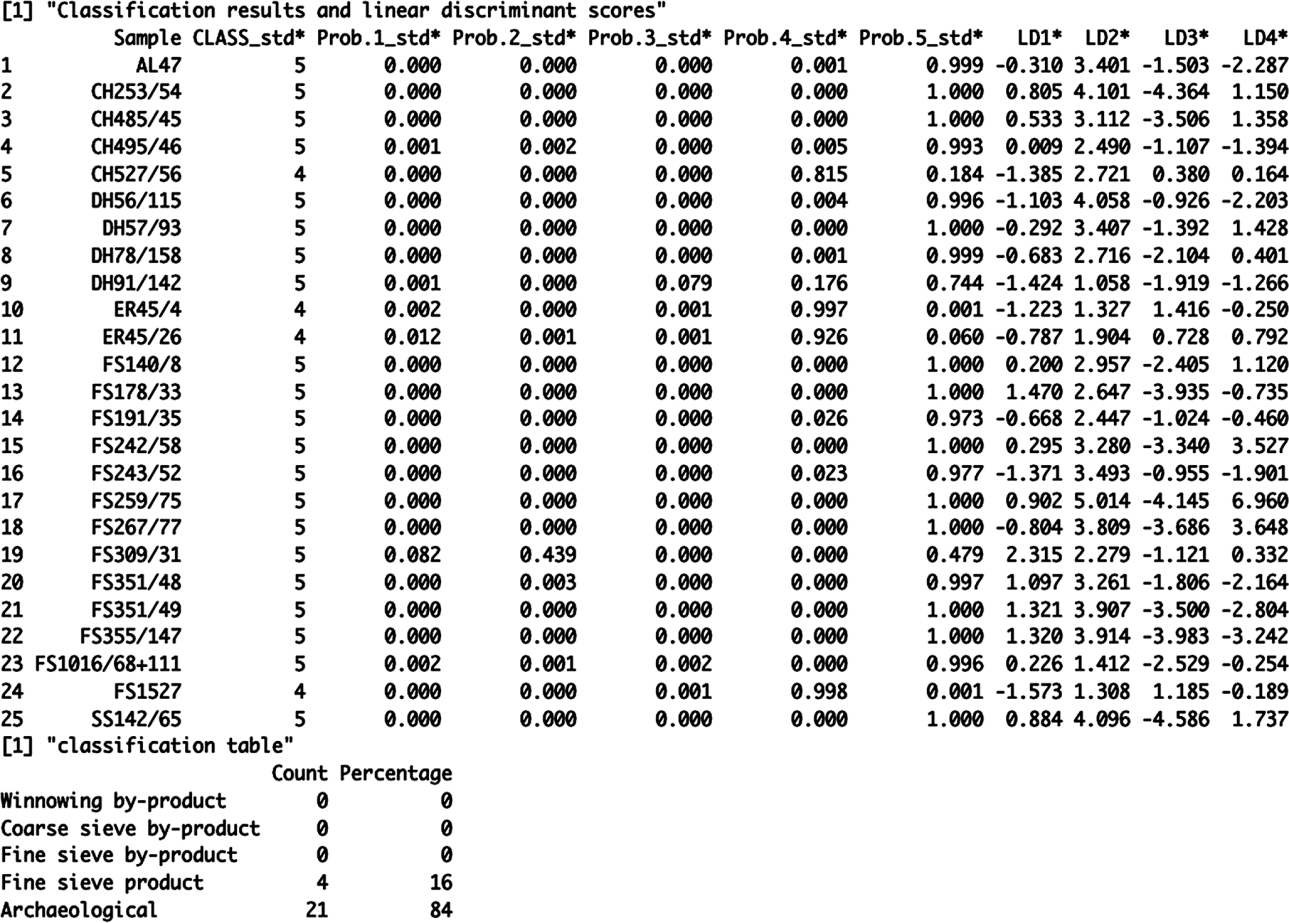
A portion of the R console output of LDA.cropplus showing the results table and classification table of the Tell Brak

**Fig. 13:**
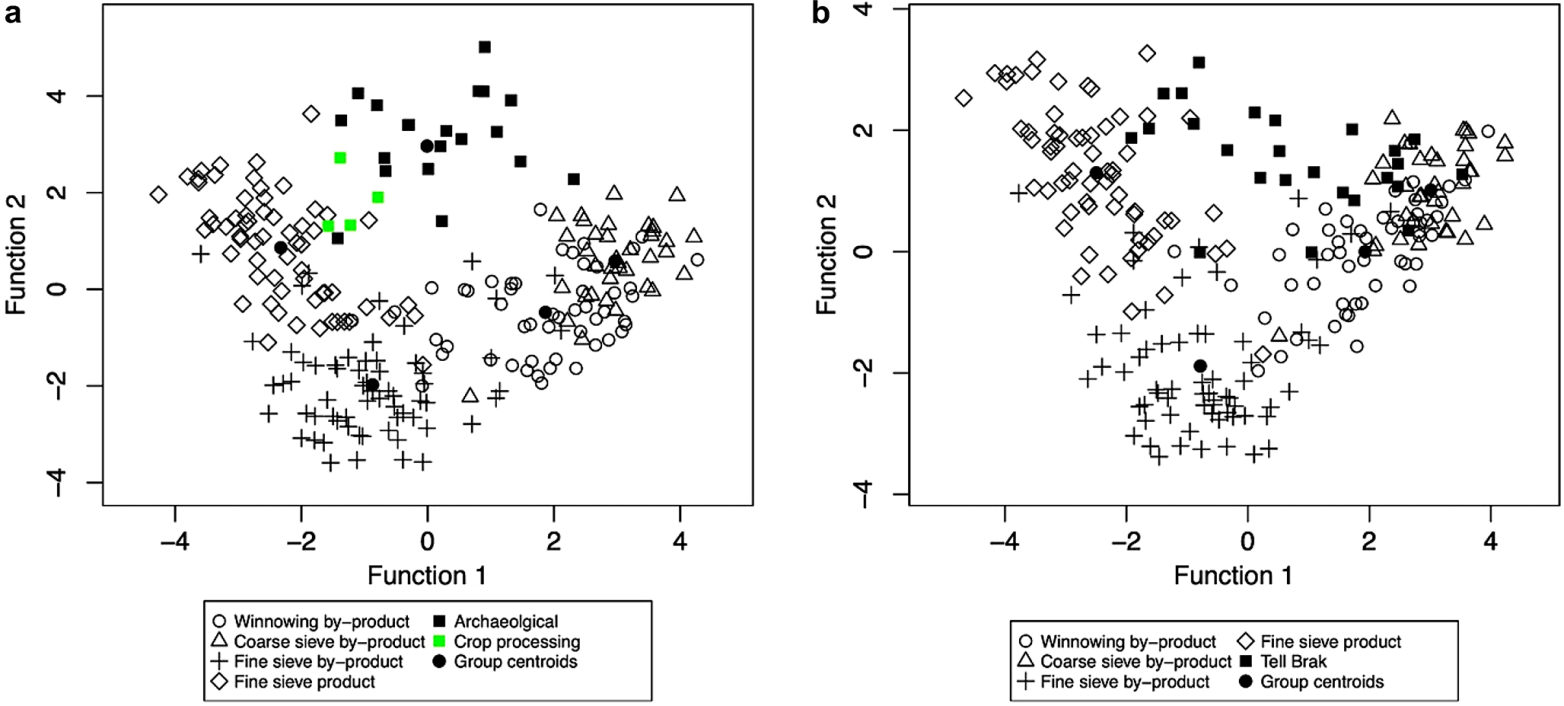
**a**, a plot of the Tell Brak data created using crop.plus_plot2D, with samples classified as a crop processing group coloured green; **b**, a plot of the Tell Brak data created using crop.plot2D

**Fig. 14:**
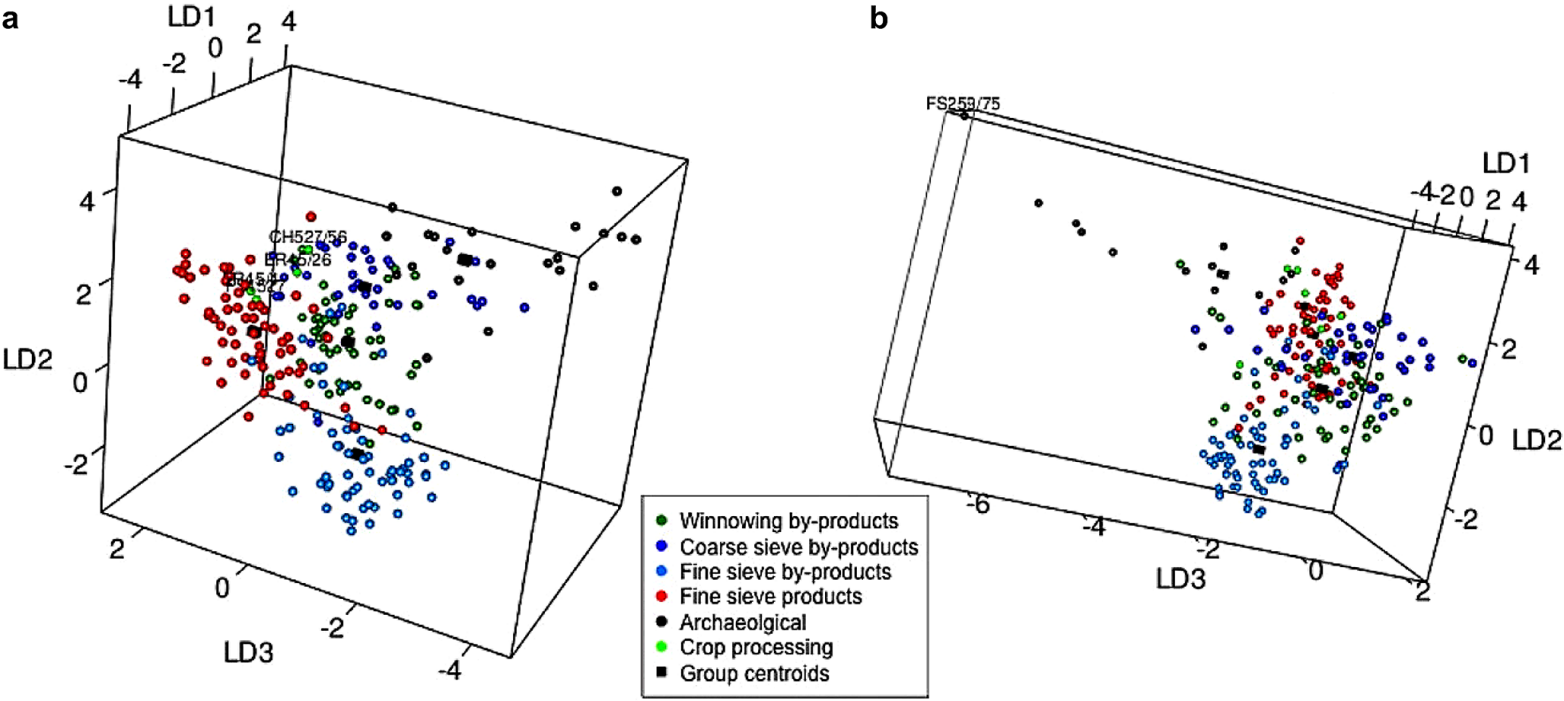
**a**, a 3D plot of the Tell Brak discriminant analysis results produced using LDAcrop.plus, showing the second and third axes with samples coloured and labelled based on classification as either archaeological or crop processing; **b**, a 3D plot of the Tell Brak discriminant analysis showing the results of LDAcrop.plus when using a reduced set of species with samples coloured and labelled based on classification as either archaeological or crop processing

LDAcrop.plus was used to analyse the Tell Brak data; the output from crop.dataorg above (i.e. 20 items etc.) was used (ESM 7: code line 109). The resultant classification table shows that 84% of the archaeobotanical samples are re-classified as archaeological rather than as one of the crop processing (by-)products (Fig. 12). The probabilities of these samples being most like group 5 are all above 90% except for two samples DH91/142 and FS309/31(Fig. 12). The four samples not classified as archaeological were CH527/56, ER45/26, ER45/4 and FS1527. These samples were classified as fine sieve product by LDAcrop.pro (see Table 4). All are barley-dominated except for CH527/56, which is free-threshing cereal dominated. CH527/56 has been mentioned above as a possible combination of by-products from early processing and hand sorting.

crop.plus_plot2D and crop.plus_plot3D can be used to plot the results of LDAcrop.plus. These functions must be used to plot the output of LDAcrop.plus, as the x and y coordinates of the ethnographic data differ when archaeobotanical data is used in the model, something the crop.plus functions are equipped to deal with. crop.plus_plot2D was used to plot the output of LDAcrop.plus with the LDA classification of the archaeobotanical samples colour coded (archaeological vs. crop processing) (Fig. 13a) (ESM 7: code line 115). Comparison of this plot with the plot from LDAcrop.pro output shows that there is slight distortion in the crop-processing pattern but that it is minimal (Fig. 13b). Colour coding the samples base on classification using crop.plus_plot3D shows how the samples classified as archaeological cluster with the ethnographic data on axis 3 – which is not shown in the 2D plot (compare Fig. 13a with Fig. 14a) (ESM 7: code lines 113–117).

As Tell Brak is located in semi-arid south-west Asia, it is possible that the samples include material from the burning of dung, thus making them deviate from the ethnographic data. The criteria Charles (1998) proposed can be used to investigate the likelihood of this through understanding the ecology/biology of weed/wild taxa, the presence of dung remains and the behaviour of wild/weed seeds compared to crop processing (see Charles 1998 for full details). While exploring such criteria is outside the scope of this paper, a set of species (Table 3), the ecologies of which suggest derivation from dung, were removed to demonstrate the iterative processes that the use of this method requires. The new dataset was rerun through the workflow, including data cleaning to remove any sample with less than 20 items and then crop.dataorg and LDAcrop.plus (Fig. 1) (ESM 7: code lines 130–144). The classifications change with the refined data, and archaeobotanical samples classified as ‘archaeological’ reduced from 84 to 69% of samples: seven samples are now classified as one of the crop processing groups. crop.plus_plot3D shows that some samples are located at a distance from the crop processing samples on the 3rd axis – in particular sample FS259/75 (Fig. 14b). This sample lacks BFH seeds and has a high number of SFL seeds (the dominant weed combination in winnowing by-product). The high amount of *Lophochloa* and other small-seeded grasses pulls this sample out. Small-seeded grasses have at some sites been linked to dung (e.g. Bogaard et al. 2021), so this provides another possible insight which could be further explored though the removal of such species and rerunning the analysis, and/or the use of other statistical methods such as correspondence analysis.

## Discussion

The use of CropPro to determine the source of samples is another tool now freely available to archaeobotanists when investigating archaeobotanical assemblages. Determining which products or by-products are represented by archaeobotanical samples is necessary, in order to recognize the biases in sample composition introduced during crop processing. These biases can then be taken into account when interpreting weed species as indicators of cultivation practices and regimes. CropPro provides a complementary statistical tool that can be run before weed ecology statistical packages such as WeedEco (Stroud et al. 2023), to ensure that crop processing biases in the weed species represented in samples have been considered before embarking on the ecological analysis of weeds as indicators of growing conditions.

The worked examples presented here have provided an insight into the scope of the R package CropPro and the variety of ways the package can be used to investigate the stage of crop processing represented within archaeobotanical samples. Moreover, the Tell Brak data shows how CropPro can be used, in conjunction with other criteria, to understand the likelihood that other taphonomic pathways such as dung burning contributed to the archaeobotanical assemblage.

Previously published crop processing analyses of archaeobotanical data have been conducted in SPSS. It should be noted that slight differences may be observed, in particular relating to the negative and positive signs for the different discriminant functions. This is because statistically whether a group, e.g. a crop-processing group, has a negative or positive linear discriminant score is arbitrary and will differ between statistical programs. Should the ethnographic dataset be used in an alternative statistical program, for ease of comparison between different programs it is necessary to explicitly state what statistical program has been used.

It is strongly recommended that the version of the R package, R, RStudio, and the crop processing dataset used are explicitly stated within the method section of outputs to facilitate reproducibility. To cite the use of the data, models and R package described in this article we suggest including a paragraph referencing all of the components. Using the Tell Brak dataset as an example, a paragraph like the one below should be included:

*The analysis followed the procedure described in Stroud et al. (this paper). The R package CropPro*, *version 1.0.0 was used (Stroud et al.*^2023^). *The Tell Brak data were plotted in comparison to the grains/rachis nodes/weed seeds ethnographic data from Jones (*1990*). The data were also classified using the discriminant analysis functions within CropPro using two models: a model constructed from the ethnographic weed attribute data*, *and a model constructed from the ethnographic weed attribute data and archaeobotanical samples (see* Jones 1984*and Charles*^1998^*for full model details*, *Stroud et al. (this paper) for the ethnographic data). R version 4.2.2*, *and RStudio version 2022.07.02*, *were used.*

## Conclusions

The R package CropPro allows archaeobotanists to compare samples against ethnographically derived proportions and weed attribute data deriving from different stages of traditional crop processing. This package allows the application of the method developed by Jones (1984), which classifies archaeobotanical samples against a discriminant model constructed of weeds derived from ethnographically collected samples of four crop processing products and by-products. Furthermore, the package provides functions which allow archaeobotanists to investigate alternative depositional pathways where the discriminant model is constructed using the ethnographic data plus the archaeobotanical data, testing the assumption that the samples necessarily represent crop processing residues (Charles 1998).

## Electronic supplementary material

Below is the link to the electronic supplementary material.

Supplementary Material 1

Supplementary Material 2

Supplementary Material 3

Supplementary Material 4

Supplementary Material 5

Supplementary Material 6

Supplementary Material 7

Supplementary Material 8

## Footnotes

1 Interactive graphs may require the installation of XQuartz software on MacOS based computers.

2 The authors aim to submit the package to CRAN in the near future. Currently the development version of the package is available on GitHub.
